# Oxygen-15 labeled CO_2_, O_2_, and CO PET in small animals: evaluation using a 3D-mode microPET scanner and impact of reconstruction algorithms

**DOI:** 10.1186/s13550-017-0335-7

**Published:** 2017-10-27

**Authors:** Genki Horitsugi, Tadashi Watabe, Yasukazu Kanai, Hayato Ikeda, Hiroki Kato, Sadahiro Naka, Mana Ishibashi, Keiko Matsunaga, Kayako Isohashi, Eku Shimosegawa, Jun Hatazawa

**Affiliations:** 1 Department of Nuclear Medicine and Tracer Kinetics, Osaka University Graduate School of Medicine, 2-2 Yamadaoka, Suita, Osaka, 565-0871 Japan; 2 Molecular Imaging in Medicine, Osaka University Graduate School of Medicine, 2-2 Yamadaoka, Suita, Osaka, 565-0871 Japan; 3 Medical Imaging Center for Translational Research, Osaka University Graduate School of Medicine, 2-2 Yamadaoka, Suita, Osaka, 565-0871 Japan

**Keywords:** PET, O-15 gas, Image reconstruction, Small animal, Steady-state method, Quantitative value

## Abstract

**Background:**

Positron emission tomography (PET) studies using ^15^O-labeled CO_2_, O_2_, and CO have been used in humans to evaluate cerebral blood flow (CBF), the cerebral oxygen extraction fraction (OEF), and the cerebral metabolic rate of oxygen (CMRO_2_) and cerebral blood volume (CBV), respectively. In preclinical studies, however, PET studies using ^15^O-labeled gases are not widely performed because of the technical difficulties associated with handling labeled gases with a short half-life. The aims of the present study were to evaluate the scatter fraction using 3D-mode micro-PET for ^15^O-labeled gas studies and the influence of reconstruction algorithms on quantitative values.

Nine male SD rats were studied using the steady state inhalation method for ^15^O-labeled gases with arterial blood sampling. The resulting PET images were reconstructed using filtered back projection (FBP), ordered-subset expectation maximization (OSEM) 2D, or OSEM 3D followed by maximum a posteriori (OSEM3D-MAP). The quantitative values for each brain region and each reconstruction method were calculated by applying different reconstruction methods.

**Results:**

The quantitative values for the whole brain as calculated using FBP were 46.6 ± 12.5 mL/100 mL/min (CBF), 63.7 ± 7.2% (OEF), 5.72 ± 0.34 mL/100 mL/min (CMRO_2_), and 5.66 ± 0.34 mL/100 mL (CBV), respectively. The CBF and CMRO_2_ values were significantly higher when the OSEM2D and OSEM3D-MAP reconstruction methods were used, compared with FBP, whereas the OEF values were significantly lower when reconstructed using OSEM3D-MAP.

**Conclusions:**

We evaluated the difference in quantitative values among the reconstruction algorithms using 3D-mode micro-PET. The iterative reconstruction method resulted in significantly higher quantitative values for CBF and CMRO_2_, compared with the values calculated using the FBP reconstruction method.

**Electronic supplementary material:**

The online version of this article (10.1186/s13550-017-0335-7) contains supplementary material, which is available to authorized users.

## Background

^15^O-labeled gases in positron emission tomography (PET) examinations have been used for the clinical evaluation of cerebral blood flow (CBF) and oxygen metabolism in patients with steno-occlusive disease of the cerebral artery. Despite the recent increase in the use of the arterial spin labeling method for magnetic resonance imaging (MRI) or perfusion imaging using computed tomography (CT) for the evaluation of CBF, PET using ^15^O-labeled gases has been the reference standard for quantitative evaluations of CBF, the oxygen extraction fraction (OEF), the cerebral metabolic rate of oxygen (CMRO_2_), and the cerebral blood volume (CBV). Among these quantitative values, oxygen utility information, such as the OEF, can only be obtained using PET with ^15^O-labeled gases, and these parameters are regarded as the best predictors of stroke recurrence [1].

In the preclinical field, PET studies using ^15^O-labeled gases are not widely performed mainly because of the technical difficulties associated with handling labeled gases with a short half-life in small animals. We have established a methodology for the quantitative evaluation of ^15^O-labeled gases using PET in small animals and have reported the CBF, OEF, CMRO_2_, and CBV of normal anesthetized rats examined using the 2D-mode of a clinical PET scanner [2]. However, recently developed microPET scanners are dedicated to 3D-mode acquisition similar to clinical PET scanners, and the scatter fraction and random coincidence can be problems for quantitative PET examinations, especially in studies involving the continuous inhalation of ^15^O-labeled gases [3, 4].

Meanwhile, iterative reconstruction algorithms, such as an ordered-subset expectation maximization (OSEM), are often used in small animal studies because they enable better image quality and spatial resolution, compared with the filtered back projection (FBP) algorithm [5, 6]. In previous studies, the spatial resolutions measured using iterative reconstruction algorithms were better than those measured using FBP, although the results depended on the parameter used for the iterative reconstruction algorithms [7–9]. OSEM2D and OSEM3D were major reconstruction methods for iterative reconstruction. In OSEM2D, 3D data is generally converted into 2D data using a rebinning step such as Fourier rebinning method to reduce the reconstruction time. In the clinical field, OSEM3D reportedly improves the image quality, compared with OSEM2D [10]. In addition, OSEM3D with point spread function correction, such as OSEM3D followed by maximum a posteriori (MAP), is used in the preclinical field and has been reported to exhibit a higher uniformity, higher recovery coefficient, and lower spill over ratio than either OSEM2D or FBP [11].

Regardless of the clinical situation and preclinical examinations, FBP is primarily selected as the reconstruction algorithm for quantitative PET measurement [2–4, 12–14]. Although FBP is a standard reconstruction method for quantitative PET examinations, the influence of iterative reconstruction algorithms on ^15^O-labeled gases studies in small animals has not been previously evaluated.

In the present study, we evaluated quantitative values using ^15^O-labeled gases and 3D-mode microPET, focusing on the scatter fraction from the lung, and examined the impact of different reconstruction algorithms on the quantitative values of CBF, OEF, CMRO_2_, and CBV.

## Methods

### Preparation of ^15^O-labeled gases

^15^O-labeled gases were produced from an N_2_ gas containing 2.0% CO_2_ (for ^15^O-CO_2_) or 2.0% O_2_ (for ^15^O-CO and ^15^O-O_2_) using an ^14^N (d, n) ^15^O reaction. The cyclotron (CYPRIS HM-12S; Sumitomo Heavy Industries Ltd., Tokyo) was operated with an average beam current of 7 μA and a deuteron acceleration energy of 6 MeV. The gas concentration stabilizing system (CYPRIS G3-A; Sumitomo Heavy Industries Ltd., Tokyo) controlled the flow rates and the radioactivity concentrations of the ^15^O-labeled gases. ^15^O-labeled gases were supplied through a gas mixture device with pure oxygen to maintain the oxygen concentration at around 30%.

### Animal preparation

The animal experiment was approved by the Institutional Animal Care and Use Committee of the Osaka University Graduate School of Medicine (Approval number: 20-144-2). Normal male Sprague-Dawley (SD) rats (Japan SLC Inc., Hamamatsu, Japan) were housed under a 12-h light and dark cycle with free access to food and water. Rats (*n* = 9, 9 weeks, body weight = 310 ± 19 g) were anesthetized by the inhalation of 2% isoflurane, and a polyethylene tube was set into the femoral artery for arterial blood sampling. The anesthesia was switched to an intramuscular injection of xylazine (4.8 μg/g of body weight), butorphanol (1.6 μg/g of body weight), and midazolam (1.2 μg/g of body weight), and the flexible plastic tube was inserted into the trachea for the inhalation of ^15^O-labeled gases after the tracheotomy. The respirator (SN-480-7; Shinano Seisakusyo) was connected to the airway tube, and artificial ventilation (Additional file 1: Figure S1) was started (respiratory rate = 60 breaths per min, tidal volume = 3 mL).

### PET-CT acquisition

The PET measurement was performed using a small-animal PET-CT scanner (Inveon MM; Siemens Medical Solutions, Knoxville, USA). The rats were placed in a supine position on the warming bed, and their rectal temperature, which was kept at 37.0 °C ± 0.5 °C, was monitored. Heart rate, systolic blood pressure (SBP), and diastolic blood pressure (DBP) were measured using a noninvasive system (BP-98A-L; Softron, Japan) by the tail-cuff method before and after each PET measurement. The PET scan was started at the same time as the start of the inhalation of each ^15^O-labeled gas. Using the steady-state method, ^15^O-labeled gases were ventilated continuously during the 16-min PET scanning period for the ^15^O-CO_2_ gas (200 MBq/min) and the ^15^O-O_2_ gas (400 MBq/min) studies (*n* = 9). In addition, ^15^O-CO gas (400 MBq/min) inhalation was also performed for 3 min, and the PET measurement was continued for up to 13 min in some animals (*n* = 6 of 9). Arterial blood samplings were performed at 13 and 16 min after the start of the PET scan in the ^15^O-CO_2_ and ^15^O-O_2_ studies and at 10 min after the start of the PET scan in the ^15^O-CO study. Arterial blood gas data (pH, PaCO_2_, PaO_2_, SaO_2_, hematocrit, and hemoglobin) were measured using a blood gas analyzer (i-STAT; FUSO Pharmaceutical Industries, Ltd., Japan) at 13 min after the start of the PET scan in the ^15^O-CO_2_ and ^15^O-O_2_ studies. The CT scan was performed for scatter and attenuation correction using a tube voltage of 80 kV and a tube current of 140 μA after PET acquisition. The weight and radioactivity count of the whole blood and blood plasma were measured using a NaI scintillation well counter (BeWell; Molecular Imaging Labo, Osaka, Japan).

### Evaluation of the influence of ^15^O-labeled gas radioactivity in the lung on the brain

After overnight fasting, ^18^F-FDG (57.2 MBq) was administered to a normal SD rat (body weight = 319.43 g) via the tail vein under 2% isoflurane anesthesia to simulate accumulation in the brain during the steady state inhalation of ^15^O-CO_2_ gas. Based on previous data for ^18^F-FDG uptake in the brain under isoflurane anesthesia in our facility, the injected dose was calculated to be equivalent to the radioactivity in the brain during the inhalation of ^15^O-CO_2_ gas. Sixty minutes later, the rat was sacrificed with deep anesthesia, and a balloon phantom was inserted into the thoracic cavity after the removal of the lung. The balloon phantom was connected to the airway tube to ventilate ^15^O-labeled gases. Each 10-min PET scan was started 2 min after the start of the supply of ^15^O-O_2_ gas with different activity concentrations (0, 200, 400, and 600 MBq/min).

### Reconstruction and data analysis

All the PET images were reconstructed using FBP, OSEM2D with 16 subsets and 4 iterations, and OSEM3D-MAP with 16 subsets, 2 iterations for OSEM3D, and 18 iterations for MAP with scatter and attenuation correction. The reconstruction parameters for iterative reconstruction were decided according to the default values provided by the manufacturer and have often been used in previous studies [7, 9, 11, 15, 16]. The requested resolution of the MAP reconstruction was set to 1.5 mm. The single scatter simulation algorithm was applied as the scatter correction [17, 18]. The image matrix and the voxel size were 128 × 128 × 159 and 0.776 × 0.776 × 0.796 mm, respectively. The energy and the timing window were 350–650 keV and 3.432 ns, respectively. Cross-calibration factors between the PET scanner and the dose calibrator were measured for each reconstruction method using a cylinder phantom of the same size as the NEMA standard rat phantom. The quantitative PET images (CBF, OEF, CMRO_2_, and CBV) were generated using the steady state inhalation method and in-house software according to the protocol described in a previous study [2]. Time activity curves were obtained by setting spherical volumes of interest (VOIs; 10 mm in diameter) over the brain on dynamic PET images (1 min × 16 frames) reconstructed using OSEM2D to check the steady state for each ^15^O-labeled gas measurement. The PET images were aligned with the template of a T_2_-weighted magnetic resonance image using the rigid registration method and PMOD software, version 3.604 (PMOD Technologies). The VOI template (W. Schiffer) was automatically placed on the brain displayed in the PET images [19]. The radioactivity counts for PET in the frontal cortex, somatosensory cortex, visual cortex, striatum, thalamus, pons, cerebellum, hippocampus, midbrain, and whole brain were obtained. The quantitative values (CBF, OEF, CMRO_2_, and CBV) in each brain region were calculated using each of the reconstruction methods (Additional file 1: Figure S2). For the rat with the lung balloon phantom in the thoracic cavity, VOIs were placed on the brain and the lung balloon phantom. The radioactivity counts were compared among the different activity concentrations of supplied ^15^O-labeled gases (0, 200, 400, and 600 MBq/min).

### Statistics

The radioactivity concentrations and quantitative values calculated using each reconstruction method were compared using a one-way repeated measures analysis of variance with Bonferroni-corrected pairwise comparisons. The quantitative values reconstructed using FBP in each brain region were compared with that in the cerebellum using a paired *t* test with Bonferroni’s correction. The cerebellum was used as a reference to understand the overall trend of the distribution in the brain according to previous studies [20, 21]. The radioactivity concentration in the whole brain was compared with that in the lung using a paired *t* test for the rat with a balloon phantom in the thoracic cavity. Statistical analyses were performed using Microsoft Excel 2013 and SPSS Statistics version 17.0 (SPSS Inc., Chicago). A *P* value of less than 0.05 was considered statistically significant.

## Results

**Fig. 1:**
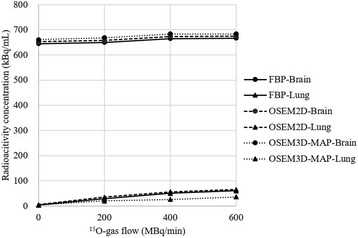
Relationship between the radioactivity of ^15^O-gas flow and the radioactivity concentrations in the brain and lung. Radioactivity in the brain showed a minor change with an increase in the radioactivity count in the lung. The percent changes were 0.7–1.1% (200 MBq/min), 3.1–3.4% (400 MBq/min), and 3.3–3.4% (600 MBq/min) compared with a control (0 MBq/min), respectively

The relationship between the radioactivity of flowing ^15^O-labeled gases and the radioactivity concentration is shown in Fig. 1 (Additional file 1: Figure S3). The radioactivity count as measured using PET was significantly lower in the lung phantom than that measured in the brain (*P* < 0.01). In all the reconstruction methods, the PET radioactivity count in the brain showed a small and minor increase compared to the count without the supply of ^15^O-labeled gases.

**Fig. 2:**
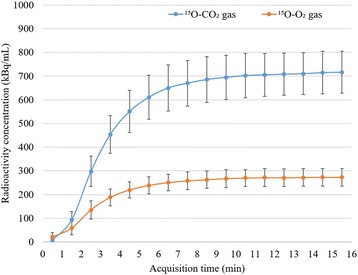
Time activity curves in the brain during the inhalation of ^15^O-CO_2_ and ^15^O-O_2_ gases. The radioactivity concentrations in the brain were obtained by setting spherical VOIs (10 mm in diameter) on the dynamic PET images. The stability of radioactivity in the brain was maintained at 10 min after the start of ^15^O-labeled gas inhalation

**Fig. 3:**
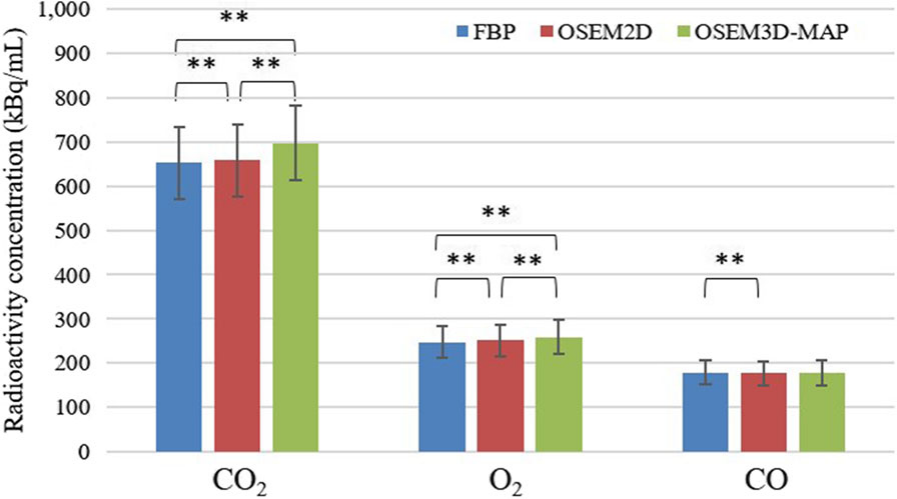
Evaluation of PET count values in whole brain obtained by each reconstruction (**P < 0.01). In the ^15^O-CO_2_ and ^15^O-O_2_ studies, the PET count values were higher in the order of OSEM3D-MAP, OSEM2D, and FBP

**Fig. 4:**
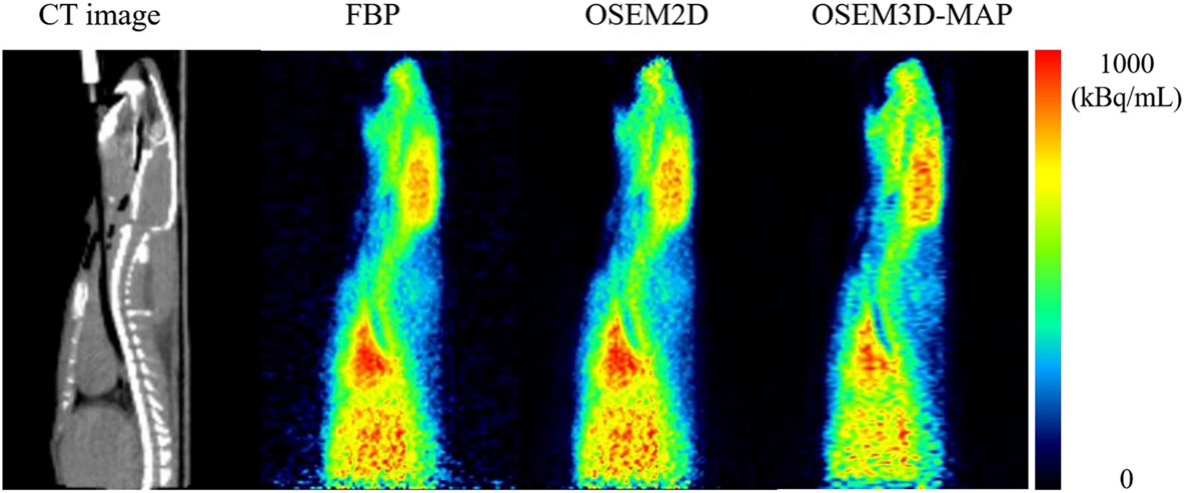
Sagittal PET images reconstructed using FBP, OSEM2D, and OSEM3D-MAP during the steady state in an ^15^O-CO_2_ study and a CT image. The PET image obtained using OSEM3D-MAP showed a higher accumulation in the brain than those images obtained using FBP and OSEM2D based on a visual assessment

The mean pH, PaCO_2_, PaO_2_, SaO_2_ hematocrit, hemoglobin concentration, heart rate, SBP, and DBP are shown in Table 1. These parameters were within physiological ranges. The stability of radioactivity was confirmed by the time activity curve in the brain during each PET measurement (Fig. 2). A comparison of the PET radioactivity counts in the whole brain is shown in Fig. 3. In both the ^15^O-CO_2_ and ^15^O-O_2_ studies, the radioactivity counts as determined using OSEM2D and OSEM3D-MAP were significantly higher than those determined using FBP (*P* < 0.01 and *P* < 0.01, respectively). In the ^15^O-CO studies, the radioactivity count value as determined using OSEM2D was significantly lower than that determined using FBP. Representative PET images during the steady state inhalation of ^15^O-CO_2_ gas are shown in Fig. 4.

**Table 1:** Arterial blood gas data, heart rate (HR), systolic blood pressure (SBP), and diastolic blood pressure (DBP) during PET measurement

Parameter	Average ± standard deviation
pH	7.40 ± 0.01
PaCO_2_ (mmHg)	44.9 ± 4.0
PaO_2_ (mmHg)	121 ± 21
SaO_2_ (%)	98 ± 1
Hct (%PCV)	39 ± 3
Hb (g/dL)	13.1 ± 0.9
HR (bpm)	317 ± 28
SBP (mmHg)	115 ± 16
DBP (mmHg)	84 ± 14

**Fig. 5:**
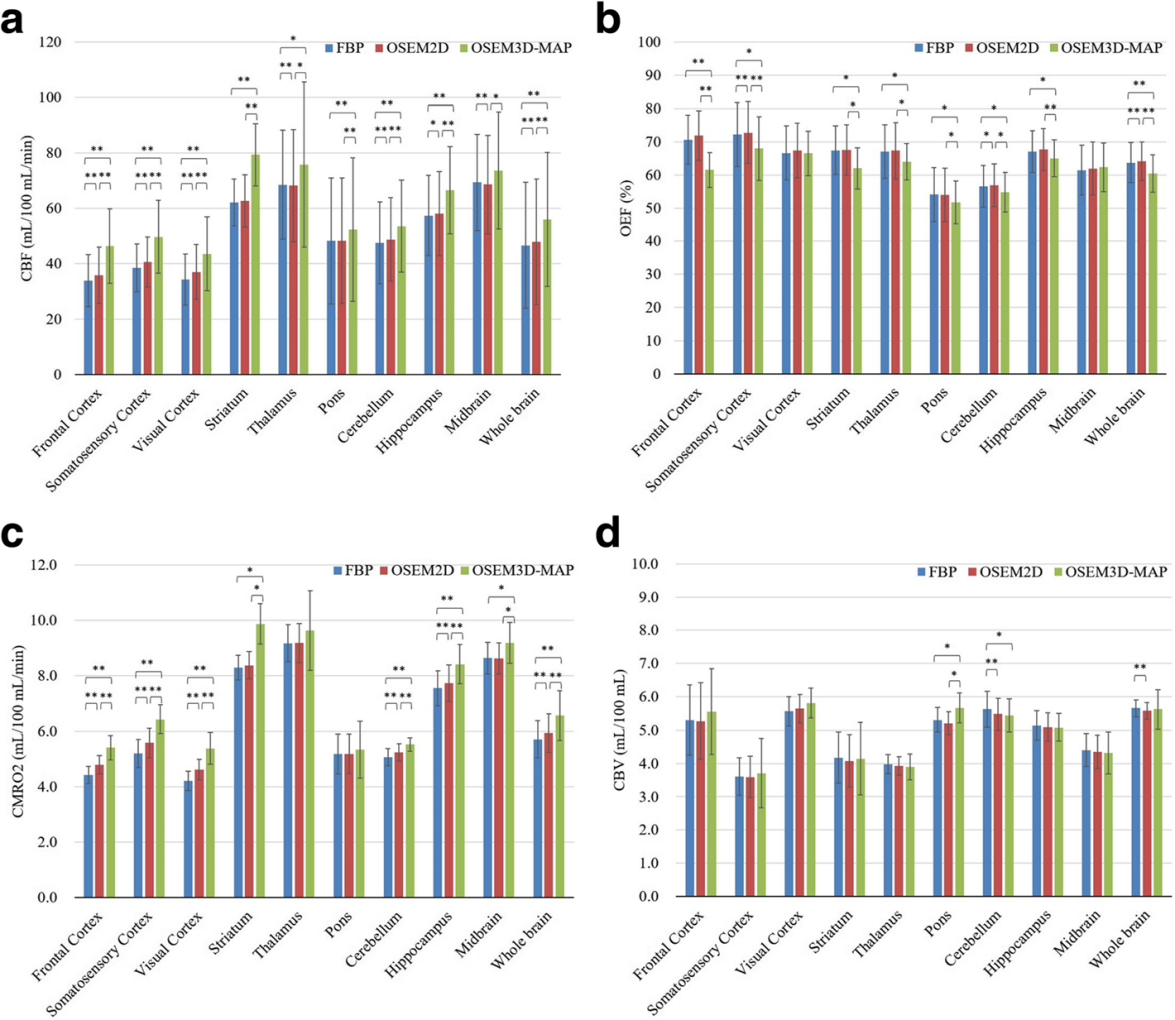
CBF (**a**), OEF (**b**), CMRO_2_ (**c**), and CBV (**d**) of each brain region according to each reconstruction method. The quantitative values obtained using each reconstruction were compared using a paired *t* test with Bonferroni’s correction (*P < 0.05, **P < 0.01). In the whole brain, OSEM3D-MAP revealed a 20% increase in CBF and a 15% increase in CMRO_2_, compared with the FBP findings, which resulted in a 5% decrease in the OEF. The differences in CBV among the three reconstruction methods were relatively small

Figure 5 and Table 2 show the quantitative values in each brain region. The CBF in the whole brain was 46.6 ± 12.5 mL/100 mL/min when reconstructed using FBP, 47.9 ± 13.2 mL/100 mL/min when reconstructed using OSEM2D, and 56.0 ± 16.3 mL/100 mL/min when reconstructed using OSEM3D-MAP. The OEF in the whole brain was 63.7 ± 7.2% when reconstructed using FBP, 64.1 ± 7.3% when reconstructed using OSEM2D, and 60.5 ± 6.0% when reconstructed using OSEM3D-MAP. The CMRO_2_ in the whole brain was 5.72 ± 0.34 mL/100 mL/min when reconstructed using FBP, 5.94 ± 0.36 mL/100 mL/min when reconstructed using OSEM2D, and 6.56 ± 0.56 mL/100 mL/min when reconstructed using OSEM3D-MAP. The CBV in the whole brain was 5.66 ± 0.34 mL/100 mL when reconstructed using FBP, 5.58 ± 0.33 mL/100 mL when reconstructed using OSEM2D, and 5.62 ± 0.37 mL/100 mL when reconstructed using OSEM3D-MAP. The CBF and CMRO_2_ values were significantly higher when the OSEM2D and OSEM3D-MAP reconstruction methods were used, compared with the results obtained using FBP reconstruction, whereas the OEF values were significantly lower when reconstructed using OSEM3D-MAP.

**Table 2:** CBF, OEF, CMRO_2_, and CBV of each brain region reconstructed using FBP (*P < 0.05, **P < 0.01 versus cerebellum)

	CBF (mL/100 mL/min)	OEF (%)	CMRO_2_ (mL/100 mL/min)	CBV (mL/100 mL)
Frontal cortex	33.9 ± 8.7^*^	70.7 ± 9.6^*^	4.44 ± 0.50	5.30 ± 0.57
Somatosensory cortex	38.4 ± 9.2^*^	72.2 ± 8.2^**^	5.21 ± 0.35	3.60 ± 0.43^**^
Visual cortex	34.2 ± 8.5^**^	66.6 ± 7.3^**^	4.21 ± 0.45^**^	5.56 ± 0.77
Striatum	62.1 ± 19.6^**^	67.5 ± 8.1^**^	8.30 ± 0.66^**^	4.17 ± 0.29^**^
Thalamus	68.5 ± 22.7^**^	67.1 ± 8.1^**^	9.17 ± 0.72^**^	3.97 ± 0.36^**^
Pons	48.2 ± 14.9	54.1 ± 6.3	5.19 ± 0.30	5.31 ± 0.53
Cerebellum	47.5 ± 14.5	56.6 ± 6.3	5.07 ± 0.63	5.63 ± 0.44
Hippocampus	57.4 ± 17.3^**^	67.1 ± 7.5^**^	7.55 ± 0.57^**^	5.14 ± 0.49
Midbrain	69.3 ± 22.7^**^	61.4 ± 6.0^*^	8.63 ± 0.67^**^	4.40 ± 0.25^**^
Whole brain	46.6 ± 12.5	63.7 ± 7.2^**^	5.72 ± 0.34^*^	5.66 ± 0.34

**Fig. 6:**
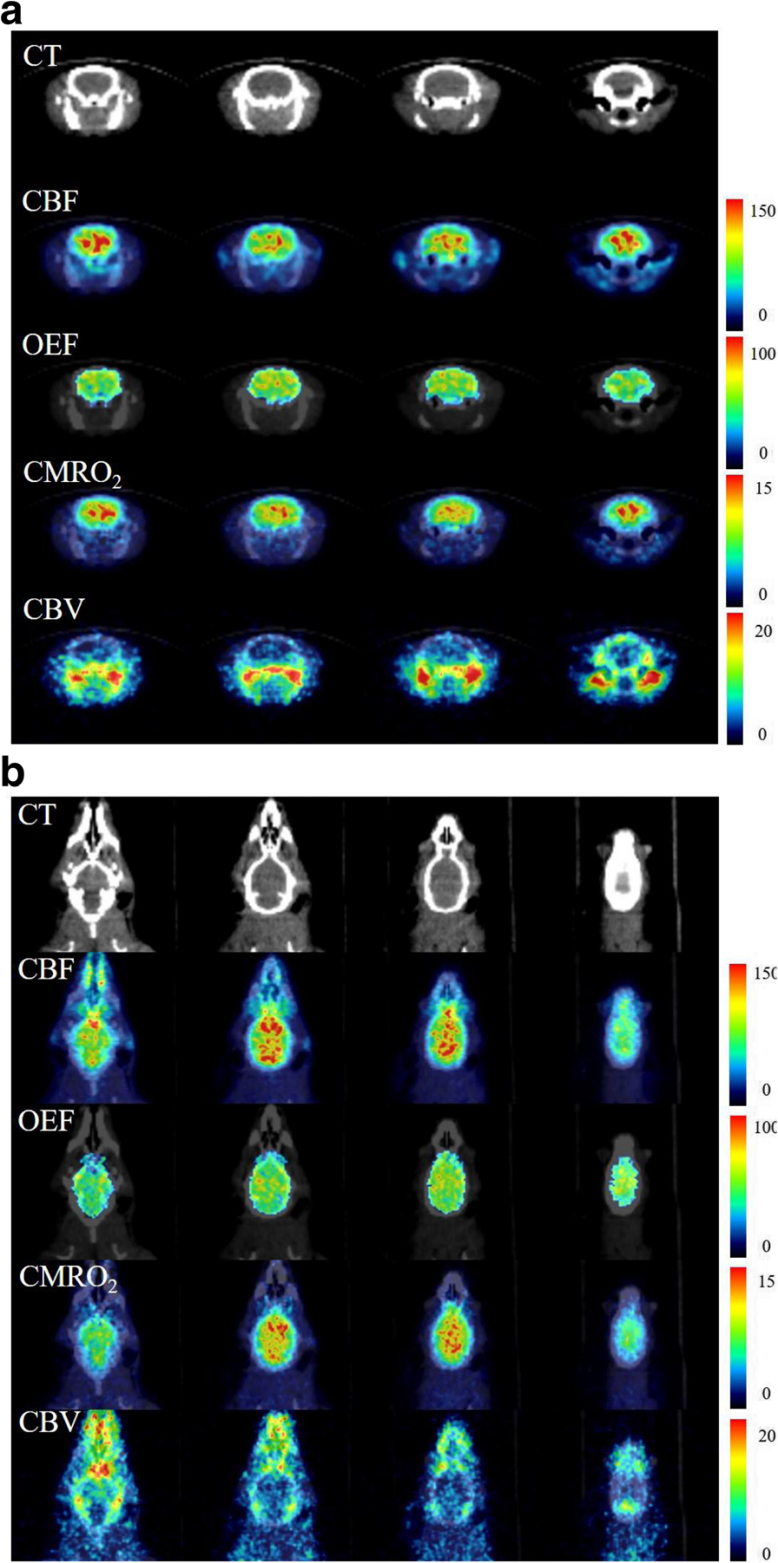
Quantitative PET images (CBF, OEF, CMRO_2_, and CBV) when reconstructed using OSEM2D and CT images. **a** Transaxial images and **b** coronal images of quantitative value in normal rats fused with CT images

Representative images of CBF, OEF, CMRO_2_, and CBV are shown in Fig. 6. Table 2 shows that a significantly higher OEF was present in the cortex region and significantly higher CBF, OEF, and CMRO_2_ values were present in the striatum, the thalamus, the hippocampus, and the midbrain, compared with the cerebellum.

## Discussion

In the present study, the primary purpose was to evaluate quantitative values using ^15^O-labeled gases and 3D-mode microPET. In 3D-mode acquisition, the scatter fractions from both inside and outside the FOV can be problematic for quantitative evaluations. The high radioactivity of ^15^O-labeled gases in the face mask, which is included in the FOV, reportedly affects the PET image in clinical situations [3, 4]. Using a clinical PET scanner, Ibaraki et al. reported the influence of random events and scatter coincidences from outside the FOV when using a 3D-mode PET scanner as well as the utility of a neck shield, which improved the noise equivalent count ratio by reducing the scatter effects [4]. Hori et al. evaluated the influence of scatter from inside and outside the FOV on PET images and demonstrated the validity of using a 3D PET scanner for ^15^O-labeled gas-inhalation PET studies [3]. When using a microPET scanner with a long axial-FOV, such as Inveon for small animals, the main scatter source is located inside the FOV. Consequently, the increase in scattered and random events because of the high radioactivity in the lung can potentially affect the quantitative values. We evaluated the influence of scattered and random events in the lung on the radioactivity in the brain and found that it was relatively small when using a 3D-mode microPET scanner with a long axial-FOV. In the lung phantom study, the increase in the radioactivity count for the whole brain was only 0.7–1.1% in the ^15^O-CO_2_ study and 3.1–3.4% in the ^15^O-O_2_ study (Additional file 1: Figure S4). Using clinical PET, quantitative studies can be performed using a 3D PET scanner even though the scatter coincidence is larger in 3D-mode than in 2D-mode [12]. The scatter fractions of clinical PET scanners have been reported for both 2D-mode and 3D-mode (13.1–21.4% and 31.8–45%, respectively) [22–24]. The scatter fraction of the small animal PET scanner that was used in the present study was 17.2% for a rat-sized phantom, which was equivalent to that of a clinical PET scanner used in the 2D-mode [25]. In another report, Konik et al. investigated the relationship between the scatter fraction and an object’s size using a Monte-Carlo-based simulation and various sizes of digital phantoms [26]. The scatter fraction ranged from 12 to 25% for rat-sized phantoms (phantom diameters of 3–9 cm) and from 26 to 46% for human-sized phantoms (phantom diameters of 10–56 cm). For the small animal PET scanner, the scatter fraction increased linearly with the phantom diameter. The scatter fraction for small animal PET studies is less than that for human studies since the subject size is very small. In quantitative studies using a small animal 3D PET scanner, the scatter fraction is roughly equivalent to that of the 2D-mode using a clinical PET scanner. In the present study, we focused on the influence of the radioactivity in the lung which was considered to be problematic in a previous clinical study. As a result, the radioactivity in the lung and the tracheal tube was found to be relatively low, as shown in Fig. 4, and uptake in the myocardium or liver was comparable to the distribution in other PET studies, such as those for FDG PET. However, the influence of high radioactivity levels in other organs should be evaluated in a future study. Figure 4 showed a higher radioactivity in the brain using OSEM3D-MAP, compared to that using FBP and OSEM2D. However, the radioactivity in the body as determined using OSEM3D-MAP was lower than the levels determined using FBP and OSEM2D. Mannheim et al. reported that differences in object size and the reconstruction method influenced the quantitative accuracy [27]. The influence arising from the difference in object size between FBP and OSEM2D was minimal, but OSEM3D and MAP showed a different tendency than that of FBP and OSEM2D. Therefore, the radioactivity in the brain and the body trunk as evaluated using OSEM3D-MAP might exhibit a different tendency from the radioactivities determined using FBP and OSEM2D.

In the present study, the CBF, CMRO_2_, and OEF images were calculated using the steady-state method, which was established by Frackowiak et al. [28]. Although the relationship was not linear, a positive correlation was observed between CBF and the ^15^O-CO_2_ PET count as well as CMRO_2_ and the ^15^O-O_2_ PET count. When either the ^15^O-CO_2_ PET count or the ^15^O-O_2_ PET count increases, the CBF or CMRO_2_ also increases. However, changes in the OEF depend on the relationship between the CBF and CMRO_2_, as the OEF is the ratio of the CMRO_2_ to CBF. For CBF as reconstructed using OSME3D-MAP, the percent differences from the values calculated using FBP were 19.6% for the whole brain, 36.7% for the frontal cortex, 28.9% for the somatosensory cortex, and 27.4% for the striatum. The differences in CBF in the deep regions of the brain (thalamus, pons, and midbrain) were relatively small, compared with those in the cortex regions. The degree of difference was smaller for CMRO_2_ than for CBF when reconstructed using OSEM3D-MAP, resulting in lower OEF values for the OSEM3D-MAP reconstructions for most regions of the brain, compared with the values calculated using FBP (percent changes − 8.42–0.31%).

In whole-body PET imaging in the field of oncology, OSEM reconstruction improves both the signal-to-noise ratio and tumor detectability [29]. Kim et al. compared reconstruction methods in a small animal cardiac PET study and concluded that OSEM3D provides better image quality and reduced image noise, compared with FBP [30]. On the other hand, Bahri et al. reported that the PET count values determined using OSEM3D were overestimated by 5%, compared with the true radioactivity values, although the PET count values determined using FBP and OSEM2D were within 99% of the true radioactivity value [31]. Weber et al. demonstrated the effect of different reconstruction algorithms on the results of a dynamic PET study [15]. They reported that the time-activity curve for a tumor as obtained using OSEM2D was consistent with that obtained using FBP; however, the curve obtained using OSEM3D-MAP was 10% higher than that obtained using FBP. The results of these reports regarding OSEM3D-MAP are similar to our results. Although OSEM3D improves tumor detectability in oncology and provides better image quality in cardiology, it may be suitable to use FBP or OSEM2D for quantitative ^15^O-gas PET studies.

In our previous study, the CBF was underestimated as 32.3 ± 4.5 mL/100 mL/min in normal anesthetized rats because of the partial volume effect arising from the use of a clinical PET scanner. Kobayashi et al. used the H_2_^15^O steady state method with a bolus and a slowly increasing injection using a multiprogrammable syringe pump and reported that the CBF was 49.2 ± 5.4 mL/100 mL/min [14]. In our study, the CBF in the whole brain was 46.6 ± 12.5 mL/100 mL/min when reconstructed using FBP, which was in close agreement with the above-mentioned report. In this study, the OEF was calculated as 63.7 ± 7.2% in the whole brain, which was consistent with our previous study (64.6 ± 9.1%). This OEF value in rats was higher than that in normal humans (44%) [32]. In our previous study, a low PaO_2_ was observed because of the low oxygen content in ^15^O-labeled gases and the effect of anesthesia [2]. In the present study, we introduced the use of a gas mixture device to supply the O_2_, and we were able to perform the PET experiments successfully while maintaining the arterial gas parameters of rats, such as PaO_2_ and PaCO_2_, within a physiological range.

The disadvantage of our quantitative PET technique using ^15^O-labeled gases was that we needed to perform an arterial blood sampling for each ^15^O-labeled gas measurement. The next challenge will be to derive input from the PET images, since the heart or large vessels were included in the PET FOV in the repetitive PET experiments. A limitation of the present study was that only a single parameter was selected for the OSEM reconstruction. Since these parameters may affect quantitative images, they should be evaluated in a future study.

## Conclusion

This study examined the use of 3D-mode micro-PET and the differences in quantitative values among various reconstruction algorithms. The iterative reconstruction method resulted in significantly higher quantitative values for CBF and CMRO_2_, compared with the FBP reconstruction method.

## Additional file

Additional file 1: Figure S1. Schema of the tube connection for the ^15^O-gas supply. **Figure S2.** Workflow for image reconstruction and data analysis. **Figure S3.** Relationship between the radioactivity of ^15^O-gas flow and the radioactivity concentration in the lung. **Figure S4.** Percent change in the radioactivity concentration against FBP (***P* < 0.01). (DOCX 312 kb)

## Abbreviations

CBF: Cerebral blood flow
CBV: Cerebral blood volume
CMRO_2_: Cerebral metabolic rate of oxygen
CT: Computed tomography
DBP: Diastric blood pressure
FBP: Filtered back projection
MAP: Maximum a posteriori
MRI: Magnetic resonance imaging
OEF: Oxygen extraction fraction
OSEM: Ordered-subset expectation maximization
PET: Positron emission tomography
SBP: Systolic blood pressure
SD: Sprague-Dawley
VOI: Volume of interest
