# Supplementary material for: Oxygen-15 labeled CO2, O2, and CO PET in small animals: evaluation using a 3D-mode microPET scanner and impact of reconstruction algorithms
Source: EJNMMI Res. 2017 Oct 27;7:91. doi: 10.1186/s13550-017-0335-7 (PMC5660010; doi:10.1186/s13550-017-0335-7)
Supplement: Additional file 1: Figure S1. — Schema of the tube connection for the 15O-gas supply. Figure S2. Workflow for image reconstruction and data analysis. Figure S3. Relationship between the radioactivity of 15O-gas flow and the radioactivity concentration in the lung. Figure S4. Percent change in the radioactivity concentration against FBP (**P < 0.01). (DOCX 312 kb) [file 13550_2017_335_MOESM1_ESM.docx]

**Oxygen-15 labeled CO_2_, O_2_ and CO PET in small animals: evaluation using a 3D-mode microPET scanner and impact of reconstruction algorithms**

Genki Horitsugi^1^; Tadashi Watabe^1,3^; Yasukazu Kanai^2,3^; Hayato Ikeda^1^; Hiroki Kato^1,3^; Sadahiro Naka^1^; Mana Ishibashi^1^; Keiko Matsunaga^2,3^; Kayako Isohashi^1,3^; Eku Shimosegawa^2,3^; Jun Hatazawa^1,3^

Department of Nuclear Medicine and Tracer Kinetics^1^, Molecular Imaging in Medicine^2^, Medical Imaging Center for Translational Reseach^3^, Osaka University Graduate School of Medicine, Japan

Corresponding author:

Jun Hatazawa, MD, PhD

2-2 Yamadaoka, Suita, Osaka 565-0871, JAPAN

TEL: +81-6-6879-3461 FAX: +81-6-6879-3469

E-Mail: hatazawa@tracer.med.osaka-u.ac.jp

Additional files


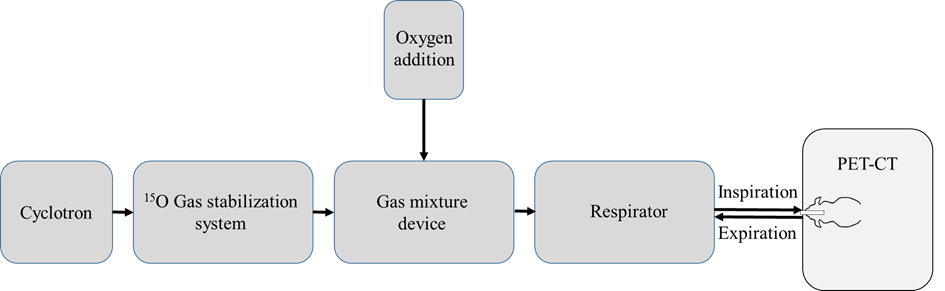


Figure S1. Schema of the tube connection for the ^15^O-gas supply.


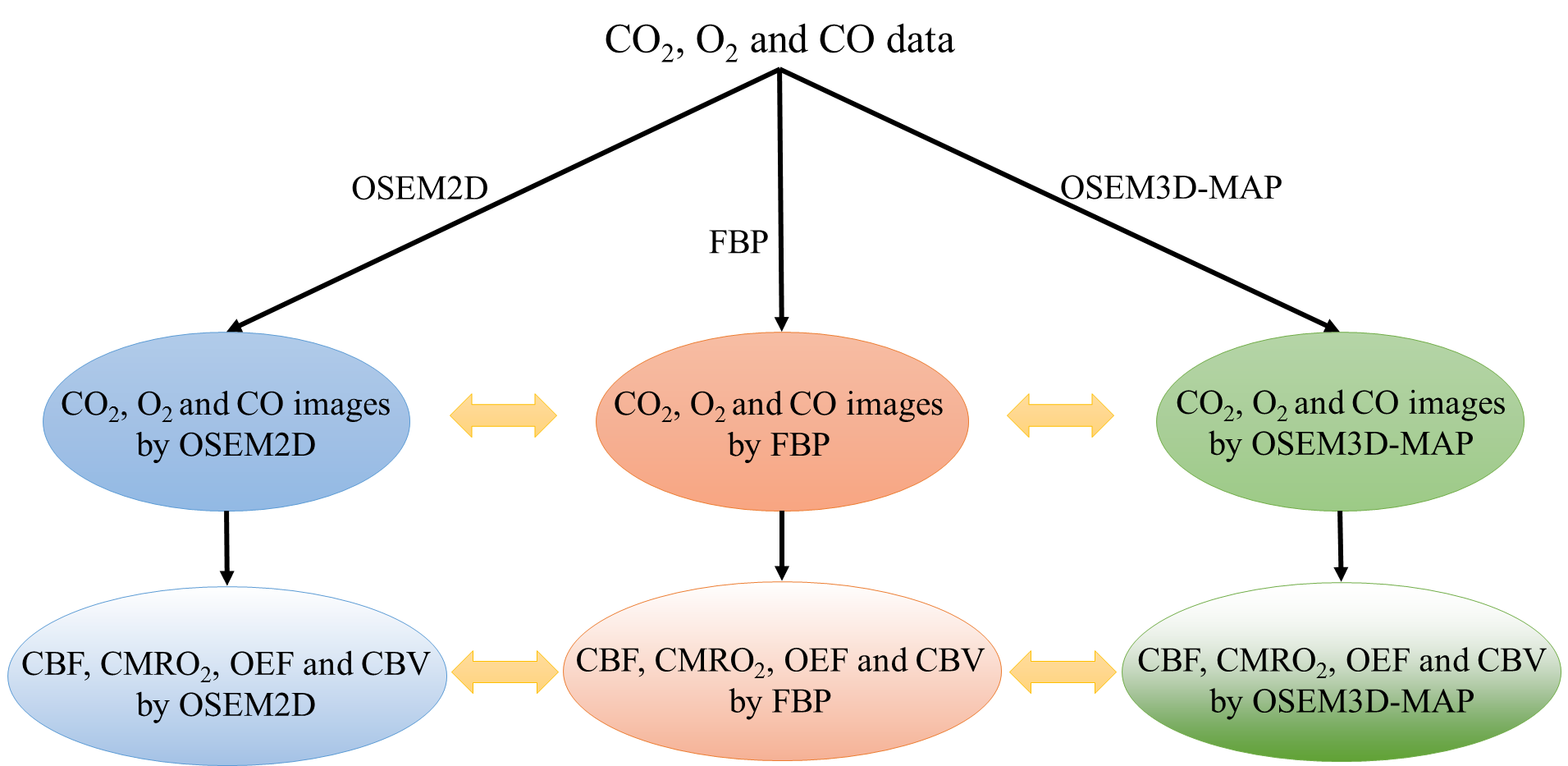


Figure S2. Workflow for image reconstruction and data analysis.


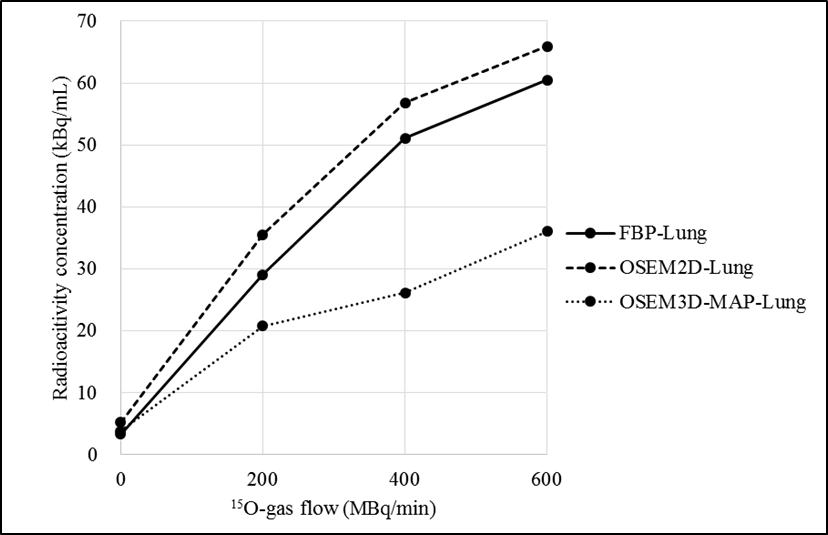


Figure S3. Relationship between the radioactivity of ^15^O-gas flow and the radioactivity concentration in the lung.


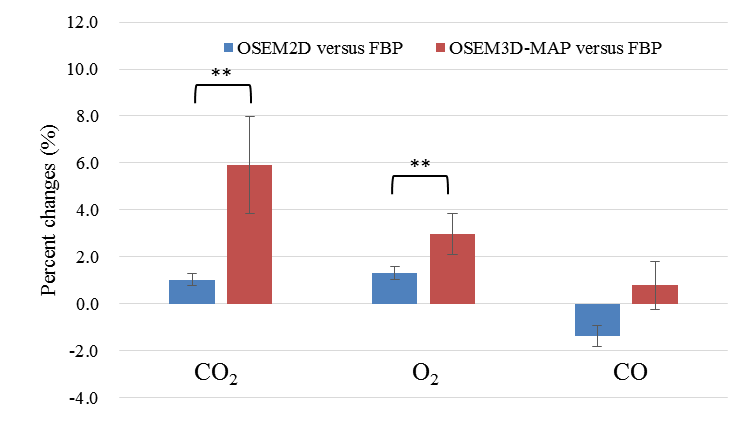


Figure S4. Percent change in the radioactivity concentration against FBP (***P* < 0.01)
